# Evaluation of multigene assays as predictors for response to neoadjuvant chemotherapy in early-stage breast cancer patients

**DOI:** 10.1038/s41523-023-00536-z

**Published:** 2023-05-06

**Authors:** Jincong Q. Freeman, Sarah Shubeck, Frederick M. Howard, Nan Chen, Rita Nanda, Dezheng Huo

**Affiliations:** 1 Department of Public Health Sciences, University of Chicago, Chicago, IL USA; 2 Department of Surgery, University of Chicago, Chicago, IL USA; 3 Department of Medicine, Section of Hematology & Oncology, University of Chicago, Chicago, IL USA

**Keywords:** Breast cancer, Predictive markers

## Abstract

OncotypeDX and MammaPrint assays have not been validated to predict pathologic complete response (pCR) to neoadjuvant chemotherapy (NACT) in early-stage breast cancer patients. We analyzed the 2010–2019 National Cancer Database and found that high OncotypeDX recurrence scores or high MammaPrint scores were associated with greater odds of pCR. Our findings suggest that OncotypeDX and MammaPrint testing predict pCR after NACT and could facilitate clinical decision-making between clinicians and patients.

The 21-gene (OncotypeDX) and 70-gene (MammaPrint) assays have been established to predict distant cancer recurrence and benefits of adjuvant therapy in early-stage breast cancer patients^1–4^ but have not been validated to predict pathologic complete response (pCR) to neoadjuvant chemotherapy (NACT). Recent small studies have reported that hormone receptor (HR)-positive and human epidermal growth factor receptor 2 (HER2)-negative breast cancer patients with a high OncotypeDX recurrence score (RS) achieve a pCR rate of 9.6%–27.5% and that there were inconsistent findings regarding the association between RS and pCR^5–10^. These studies also did not assess the shape of the relationship between OncotypeDX RS and pCR rate and, therefore, cannot determine an RS threshold at which patients are likely to achieve pCR to aid in clinical decision-making. Previous OncotypeDX RS cutoffs were established using distant metastatic recurrence as the clinical endpoint. Meanwhile, no studies to date have investigated the association between MammaPrint results and responses to NACT. With the increased use of NACT in practice, it is imperative to assess the efficacy of these multigene assays in predicting pCR, which can help inform subsequent treatment and anticipated response to therapy. Using a large clinical oncology database, we examined the ability of the OncotypeDX and MammaPrint assays to predict the likelihood of pCR after NACT among early-stage breast cancer patients.

In the OncotypeDX cohort, a total of 2219 patients received NACT at 630 institutions across the U.S. (Supplementary Table 1). Differences in race/ethnicity, progesterone receptor (PR) status, AJCC staging, and tumor grade were observed between low and high RS groups (Supplementary Table 2). The mean RS was 42.5 (SD, 15.5) in patients who achieved pCR, compared to 27.9 (SD, 13.7) in patients who did not (Table 1). There was a significant monotonic increasing trend of pCR rate by continuous RS (Fig. 1). We predicted that patients with an OncotypeDX RS of 37 were likely to have achieved a 10.0% pCR rate after NACT. The discriminating capacity of OncotypeDX was moderate to strong (AUC, 0.767; 95% CI: 0.729–0.805). Further, the relationship between pCR rates and RS was similar for young (aged ≤50 years) and old (aged >50 years) patients (Supplementary Fig. 1). Of 1181 patients with a high RS, 11.2% achieved pCR, while only 1.6% of 867 patients with a low RS did (Table 1). After adjusting for age, race/ethnicity, PR status, T stage, N stage, and tumor grade, having a high RS was associated with greater odds of pCR after NACT (adjusted odds ratio [AOR], 4.48; 95% CI: 2.44–8.22; *P* < 0.0001) (Table 1).

**Table 1:** Prediction of pathologic complete response to neoadjuvant chemotherapy in breast cancer patients who received multigene assays.

	Pathologic complete response		Logistic regression			
Variable	Not achieved	Achieved	OR (95% CI)	*P* value	AOR (95% CI)	*P* value
	No. (row %)	No. (row %)				
*Oncotype DX recurrence score*^a^						
Continuous, Mean (SD)	27.9 (13.7)	42.5 (15.5)	1.80 (1.61–2.00)^b^	<0.0001	1.58 (1.38–1.80)^b,c^	<0.0001
Categorical^d^						
0–25	853 (98.4)	14 (1.6)	1.0 (reference)		1.0 (reference)	
26–100	1049 (88.8)	132 (11.2)	7.67 (4.39–13.4)	<0.0001	4.48 (2.44–8.22)^c^	<0.0001
*MammaPrint result*						
Any early-stage breast cancer						
Low risk	>198 (>95.2)	<10 (<4.8)^g^	1.0 (reference)		1.0 (reference)	
High risk	947 (83.0)	194 (17.0)	4.53 (2.28–8.99)	<0.0001	2.21 (1.02–4.77)^e^	0.04
Hormone receptor-positive						
Low risk	>190 (>95.0)	<10 (<5.0)	1.0 (reference)		1.0 (reference)	
High risk	778 (88.2)	104 (11.8)	3.21 (1.54–6.70)	0.002	2.25 (0.99–5.15)^f^	0.05

*SD* standard deviation, *OR* odds ratio, *CI* confidence interval, *AOR* adjusted odds ratio, *PR* progesterone receptor, *ER* estrogen receptor, *HER2* human epidermal growth factor receptor 2.

^a^The Oncotype DX cohort included patients with hormone receptor (HR)-positive/HER2-negative stage I–III disease.

^b^Odds ratio per 10 unit increase in Oncotype DX score.

^c^Adjusted for age, race/ethnicity, PR status, clinical T and N stages, and tumor grade.

^d^Oncotype DX recurrence score was dichotomized per the TAILORx trial cutoff.

^e^Adjusted for age, race/ethnicity, PR status, ER status, HER2 status, clinical T and N stages, and tumor grade.

^f^Adjusted for age, race/ethnicity, PR status, HER2 status, clinical T and N stages, and tumor grade.

^g^As per NCDB requirement to protect the confidentiality of patients, we suppressed reporting of frequencies <10.

**Fig. 1:**
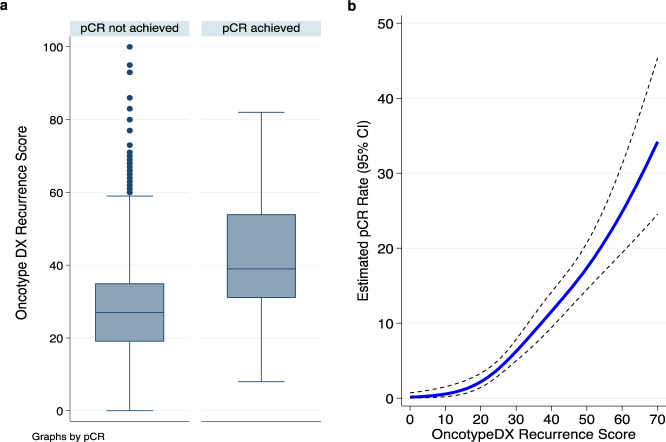
Relationship between Oncotype DX recurrence score and pCR to neoadjuvant chemotherapy in HR-positive/HER2-negative breast cancer patients. **a** Boxplot of Oncotype DX recurrence score by pCR. The horizontal lines in the box denote the first quartile, median, and third quartile. The boundaries of the whiskers are based on the 1.5 interquartile values. **b** Estimated rate of pCR using restricted cubic spline logistic regression. The two black dash lines represent the 95% confidence interval of the estimated pCR rate. Abbreviations: pCR pathologic complete response, HR hormone receptor, HER2 human epidermal growth factor receptor 2, CI confidence interval.

In the MammaPrint cohort, a total of 1349 patients treated at 337 institutions received NACT (Supplementary Table 1). There were differences in PR status, estrogen receptor (ER) status, HER2 status, and tumor grade between low-risk and high-risk groups (Supplementary Table 3). Of 1141 patients with high scores, 17.0% achieved pCR, compared to <4.8% of 208 patients with low scores (Table 1). After adjusting for age, race/ethnicity, PR status, ER status, HER2 status, T stage, N stage, and tumor grade, high scores were associated with greater odds of having achieved pCR after NACT (AOR, 2.21; 95% CI: 1.02–4.77; *P* = 0.04) (Table 1). A similar association between MammaPrint result and pCR was observed in the subset of patients with HR-positive tumors (AOR, 2.25; 95% CI: 0.99–5.15; *P* = 0.05; Table 1).

Additionally, in the OncotypeDX cohort, 64.5% of the patients with a high RS underwent a lumpectomy, while 46.5% of the patients with a low RS did. After adjusting for T stage and N stage, having a high RS was associated with greater odds of having undergone a lumpectomy (AOR, 1.83; 95% CI: 1.48–2.25; *P* < 0.0001) (Supplementary Table 4). In the MammaPrint cohort, 63.4% of the patients with a high score underwent a lumpectomy, and 51.9% of the patients with a low score did. After adjusting for the T stage and N stage, the high-risk group was associated with greater odds of having undergone a lumpectomy (AOR, 1.46; 95% CI: 1.02–2.08; *P* = 0.037) (Supplementary Table 4).

We found that one in 9 patients with an OncotypeDX RS of ≥26 achieved pCR after NACT and that high RS was associated with 7.7 times greater odds of pCR. After adjusting for clinicopathological factors, the association remained significant. These findings are consistent with previous studies^5,6^. Pease et al. reported a 9.6% pCR rate in patients with a high RS and an odds ratio of 4.9, comparing high with intermediate RS^5^. Pardo et al. found an axillary pCR rate of 27.5% after NACT in node-positive patients with a high RS but were not able to assess the association between OncotypeDX RS and pCR due to a small sample size^6^. Both studies used Genomic Health’s cutoffs (<18, 18–30, and >30), while our study used TAILORx trial cutoffs. In addition, these studies did not analyze numeric OncotypeDX values; whereas our study found that the likelihood of pCR after NACT increased monotonically as the RS increased and indicated a numerical threshold of 37 for a pCR rate of at least 10%. This finding is in concordance with a 2020 study conducted by Soliman et al, in which they calculated a 21-gene expression score using microarray datasets and showed a monotonic correlation with the pCR rate^11^. Collectively, these findings suggest that OncotypeDX RS predicts pCR in HR-positive/HER2-negative breast cancer patients who receive NACT. Given the increasing OncotypeDX utilization^12^, our findings may help clinicians tailor treatment in patients receiving NACT.

To our knowledge, this study is the first to examine the predictive capability of the MammaPrint assay on response to NACT among early-stage breast cancer patients. We found that one in 6 patients with high scores achieved pCR after NACT, and these patients were more than 4-fold as likely as those with low scores to have achieved pCR. The association remained significant after adjusting for clinicopathological factors. We also demonstrated an 11.8% rate of pCR in the subset of patients with HR-positive tumors and a similar association between MammaPrint result and pCR. Therefore, our findings indicate that MammaPrint results are predictive of pCR rates among patients who receive NACT, regardless of HR status, which may be helpful in guiding neoadjuvant systemic treatment for patients.

We also found that in both OncotypeDX and MammaPrint cohorts, more than 60% of the patients with a high score received a lumpectomy. In the OncotypeDX cohort, patients with a high RS were more likely to have the lower-stage disease—but even after adjusting for T stage and N stage, patients with a high OncotypeDX RS or a high MammaPrint score were still more than 1.5 times as likely as patients with a low OncotypeDX RS or a low MammaPrint score to have received a lumpectomy. These findings suggest that multigene assay testing might inform the likelihood of successfully downstaging tumors to allow for less invasive surgical treatment, although further study is needed, given the limitations of data available in the NCDB.

Our study is the largest to evaluate multigene assays and pCR but has several limitations. This is a retrospective study using a national oncology database that does not collect specific NACT regimen information. Thus, we cannot assess the differential impact of varying NACT regimens. Second, OncotypeDX and MammaPrint have traditionally been used to test surgical resection specimens in the adjuvant setting. However, previous studies reported a moderate-to-strong concordance (ranging from 72% to 91%) of these multigene assays between core needle biopsy and surgical specimens^13,14^. Lastly, large prospective studies with planned NACT and multigene assay testing are needed to confirm our findings.

In conclusion, OncotypeDX and MammaPrint scores were independently associated with the likelihood of pCR after NACT for early-stage breast cancer. These findings suggest a potential role for OncotypeDX or MammaPrint testing as a predictive biomarker in the neoadjuvant setting and could facilitate clinical decision-making between clinicians and patients.

## Methods

### Study design and population

We conducted an analysis of historical data from a prospective cohort of patients diagnosed with early-stage breast cancer. We obtained the data from the 2010-2019 National Cancer Database (NCDB), a joint project of the Commission on Cancer of the American College of Surgeons and the American Cancer Society^15^, which is a hospital-based cancer registry capturing approximately 72% of U.S. new cancer diagnoses annually^16^. This study with de-identified data was exempt from the University of Chicago Institutional Review Board oversight.

All patients who received NACT and had pathologic response data and OncotypeDX or MammaPrint results were eligible. Of the patients having received NACT, we further limited to those who started NACT for at least 30 days prior to their surgeries. The OncotypeDX cohort included HR-positive/HER2-negative stage I–III patients, while the MammaPrint cohort included both HR-positive and HR-negative stage I-III patients based on previous research in the adjuvant setting^3,4^. OncotypeDX RS was assessed as a categorical variable, classified as low (0–25) and high (26–100) per the TAILORx trial^17^. Because TAILORx trial cutoffs were used to predict the rate of distant recurrence and to determine an optimal threshold of RS for predicting the pCR rate, we also analyzed OncotypeDX as a continuous variable. MammaPrint results were assessed as a dichotomous variable (low risk and high risk) because numeric values were unavailable. pCR was defined as ypT0/Tis ypN0 per AJCC staging.

### Statistical analysis

Patient characteristics were summarized using descriptive statistics (mean [SD] for continuous variables and frequencies [%] for categorical variables). Restricted cubic spline logistic regression was used to explore the shape of the relationship between pCR and continuous OncotypeDX RS. The discriminating capacity of OncotypeDX was measured using the area under the ROC curve (AUC). Multivariable logistic regression models were used to examine the association between pCR and OncotypeDX or MammaPrint results, respectively. A two-sided *p*-value < 0.05 was considered statistically significant. All analyses were performed using Stata 17 (StataCorp).

### Reporting summary

Further information on research design is available in the Nature Research Reporting Summary linked to this article.

## Supplementary information

Supplementary Information

Reporting Summary

## Data Availability

Data for this study were obtained from the National Cancer Data Base (NCDB), which can be requested by submitting an application to the American College of Surgeons (ACS) at https://www.facs.org/quality-programs/cancer-programs/national-cancer-database.

## Code availability

All analyses were performed using Stata. Code can be made available from the corresponding author on reasonable request.
