# Supplementary material for: Evaluation of multigene assays as predictors for response to neoadjuvant chemotherapy in early-stage breast cancer patients
Source: NPJ Breast Cancer. 2023 May 6;9:33. doi: 10.1038/s41523-023-00536-z (PMC10164191; doi:10.1038/s41523-023-00536-z)
Supplement: Supplementary file 1 — Supplementary Information [file 41523_2023_536_MOESM1_ESM.pdf]

Supplementary Information

Supplementary Table 1. Characteristics of breast cancer patients who received neoadjuvant chemotherapy by multigene assay

Supplementary Table 2. Characteristics of HR-positive/HER2-negative breast cancer patients who received neoadjuvant chemotherapy by Oncotype DX recurrence score

Supplementary Figure 1. Estimated rates of pathologic complete response by age group using restricted cubic spline logistic regression

Supplementary Table 3. Characteristics of early-stage breast cancer patients who received neoadjuvant chemotherapy by MammaPrint result

Supplementary Table 4. Association between type of surgery and multigene assay results among early-stage breast cancer patients who received neoadjuvant chemotherapy

Supplementary Table 1. Characteristics of breast cancer patients who received neoadjuvant chemotherapy by multigene assay

**Variable**	**Oncotype DX** [a] (N=2,219), No. (%)	**MammaPrint** [b] (N=1,349), No. (%)
**Age at diagnosis**, mean (SD)	54.3 (11.7)	53.3 (11.9)
**Sex**		
Male	17 (0.8)	11 (0.8)
Female	2,202 (99.2)	1,338 (99.2)
**Race/Ethnicity** [c]		
White	1,667 (75.1)	1,002 (74.3)
Black	263 (11.9)	148 (11.0)
Asian	103 (4.6)	63 (4.7)
Hispanic	143 (6,4)	111 (8.2)
Other/unknown	43 (1.9)	25 (1.9)
**Primary payer at diagnosis**		
Uninsured	57 (2.6)	27 (2.0)
Private/managed care	1,452 (65.4)	884 (65.5)
Medicaid	189 (8.5)	134 (9.9)
Medicare	472 (21.3)	269 (19.9)
Other government/unknown	49 (2.2)	35 (2.6)
**Year of diagnosis**		
2010	86 (3.9)	33 (2.5)
2011	116 (5.2)	48 (3.6)
2012	110 (5.0)	78 (5.8)
2013	153 (6.9)	141 (10.5)
2014	199 (9.0)	153 (11.3)
2015	235 (10.6)	136 (10.1)
2016	307 (13.8)	114 (8.5)
2017	296 (13.3)	142 (10.5)
2018	324 (14.6)	187 (13.9)
2019	393 (17.7)	317 (23.5)
**Charlson-Deyo Score**		
0	1,944 (87.6)	1,193 (88.4)
1	206 (9.3)	132 (9.8)
≥2	69 (3.1)	24 (1.8)
**Histologic type**		
Ductal	1,826 (82.3)	1,128 (83.6)
Lobular	235 (10.6)	106 (7.9)
Ductal and lobular	93 (4.2)	49 (3.6)
Other	65 (2.9)	66 (4.9)
**PR status**		
Negative	420 (18.9)	469 (34.9)
Positive	1,797 (81.1)	876 (65.1)
**ER status**		
Negative	NA	307 (22.8)
Positive	2,211 (99.7)	1,042 (77.2)
**HER2 status**		
Negative	2,219 (100.0)	1,103 (81.8)
Positive	NA	246 (18.2)
**AJCC stage group**		
IA	318 (14.4)	177 (13.2)
IB	161 (7.3)	105 (7.8)
IIA	996 (45.0)	527 (39.2)

IIB	438 (19.8)	293 (21.8)
IIIA	160 (7.2)	131 (9.7)
IIIB	116 (5.2)	83 (6.2)
IIIC	27 (1.2)	30 (2.2)
**AJCC clinical T stage**		
cT1	445 (20.1)	273 (20.3)
cT2	1,290 (58.3)	781 (57.9)
cT3	355 (16.0)	223 (16.5)
cT4	123 (5.6)	71 (5.3)
**AJCC clinical nodal status**		
Negative (cN0)	1,543 (69.9)	823 (61.1)
Positive (cN1+)	664 (30.1)	523 (38.9)
**Tumor grade**		
1	218 (10.2)	101 (7.8)
2	1,153 (54.0)	588 (45.3)
3	765 (35.8)	609 (46.9)
**Receipt of neoadjuvant hormone therapy**		
No	1,485 (74.8)	790 (82.2)
Yes	501 (25.2)	164 (17.2)
**Facility type**		
Community cancer program	115 (5.9)	69 (6.0)
Comprehensive community cancer program	767 (39.1)	508 (44.0)
Academic/research program	655 (33.4)	338 (29.3)
Integrated network cancer program	425 (21.7)	240 (20.8)

Abbreviations: SD, standard deviation; ER, estrogen receptor; PR, progesterone receptor; HER2, human epidermal growth factor receptor 2; AJCC, American Joint Committee on Cancer.

[a] The Oncotype DX cohort included patients with hormone receptor-positive/HER2-negative stage I-III disease.

[b] The MammaPrint cohort included both HR-positive and HR-negative stage I-III patients.

[c] Other/unknown includes American Indian, Alaska Native, Hawaiian and other Pacific Islander, other or unknown races/ethnicities.

Supplementary Table 2. Characteristics of HR-positive/HER2-negative breast cancer patients who received neoadjuvant chemotherapy by Oncotype DX recurrence score

	Oncotype DX [a]		
**Variable**	**RS 0-25** (n=867), No. (row %)	**RS 26-100** (n=1,181), No. (row %)	***P* value** [b]
**Age at diagnosis**, mean (SD)	54.2 (11.5)	54.6 (11.9)	.48
**Race/Ethnicity** [c]			
White	677 (43.9)	865 (56.1)	.01
Black	81 (33.5)	161 (66.5)	
Asian	43 (43.4)	56 (56.6)	
Hispanic	55 (44.0)	70 (56.0)	
Other/unknown	11 (27.5)	29 (72.5)	
**Year of diagnosis**			
2010	35 (50.0)	35 (50.0)	<.0001
2011	52 (51.0)	50 (49.0)	
2012	46 (47.4)	51 (52.6)	
2013	58 (41.7)	81 (58.3)	
2014	92 (51.7)	86 (48.3)	
2015	108 (50.9)	104 (49.1)	
2016	118 (41.6)	166 (58.5)	
2017	114 (40.1)	170 (59.9)	
2018	115 (37.7)	190 (62.3)	
2019	129 (34.2)	248 (65.8)	
**Charlson-Deyo Score**			
0	765 (42.8)	1,024 (57.2)	.59
1	77 (39.5)	118 (60.5)	
≥2	25 (39.1)	39 (60.9)	
**Histologic type**			
Ductal	632 (37.5)	1,054 (62.5)	<.0001
Lobular	157 (73.0)	58 (27.0)	
Ductal and lobular	47 (55.3)	38 (44.7)	
Other	31 (50.0)	31 (50.0)	
**PR status**			
Negative	65 (16.2)	337 (83.8)	<.0001
Positive	801 (48.7)	843 (51.3)	
**AJCC stage group**			
I	180 (40.8)	261 (59.2)	.001
II	539 (40.6)	788 (59.4)	
III	148 (52.9)	132 (47.1)	
**AJCC clinical T stage**			
cT1	163 (40.6)	239 (59.5)	<.0001
cT2	457 (38.0)	745 (62.0)	
cT3	179 (55.4)	144 (44.6)	
cT4	66 (57.4)	49 (42.6)	
**AJCC clinical nodal status**			
Negative (cN0)	566 (39.6)	862 (60.4)	<.0001
Positive (cN1+)	294 (48.4)	314 (51.6)	
**Tumor grade**			
1	142 (71.4)	57 (28.6)	<.0001
2	549 (51.9)	509 (48.1)	
3	134 (18.7)	583 (81.3)	
**Days from initial diagnosis to start of chemotherapy**, median (IQR)	44 (30-63)	44 (34-61)	.47

**Receipt of neoadjuvant hormone therapy**			
No	555 (40.5)	814 (59.5)	<.0001
Yes	250 (53.3)	219 (46.7)	

Abbreviations: HR, hormone receptor; HER2, human epidermal growth factor receptor 2; RS, recurrence score; SD, standard deviation; PR, progesterone receptor; AJCC, American Joint Committee on Cancer.
[a] Oncotype DX recurrence score was dichotomized per the TAILORx trial cutoff.
[b] *P* values were calculated using Student's t or Wilcoxon rank-sum tests for continuous variables and Pearson's Chi-Squared or Fisher's exact tests for categorical variables.
[c] Other/unknown includes American Indian, Alaska Native, Hawaiian and other Pacific Islander, other or unknown races/ethnicities.

Supplementary Figure 1. Estimated rates of pathologic complete response by age group using restricted cubic spline logistic regression

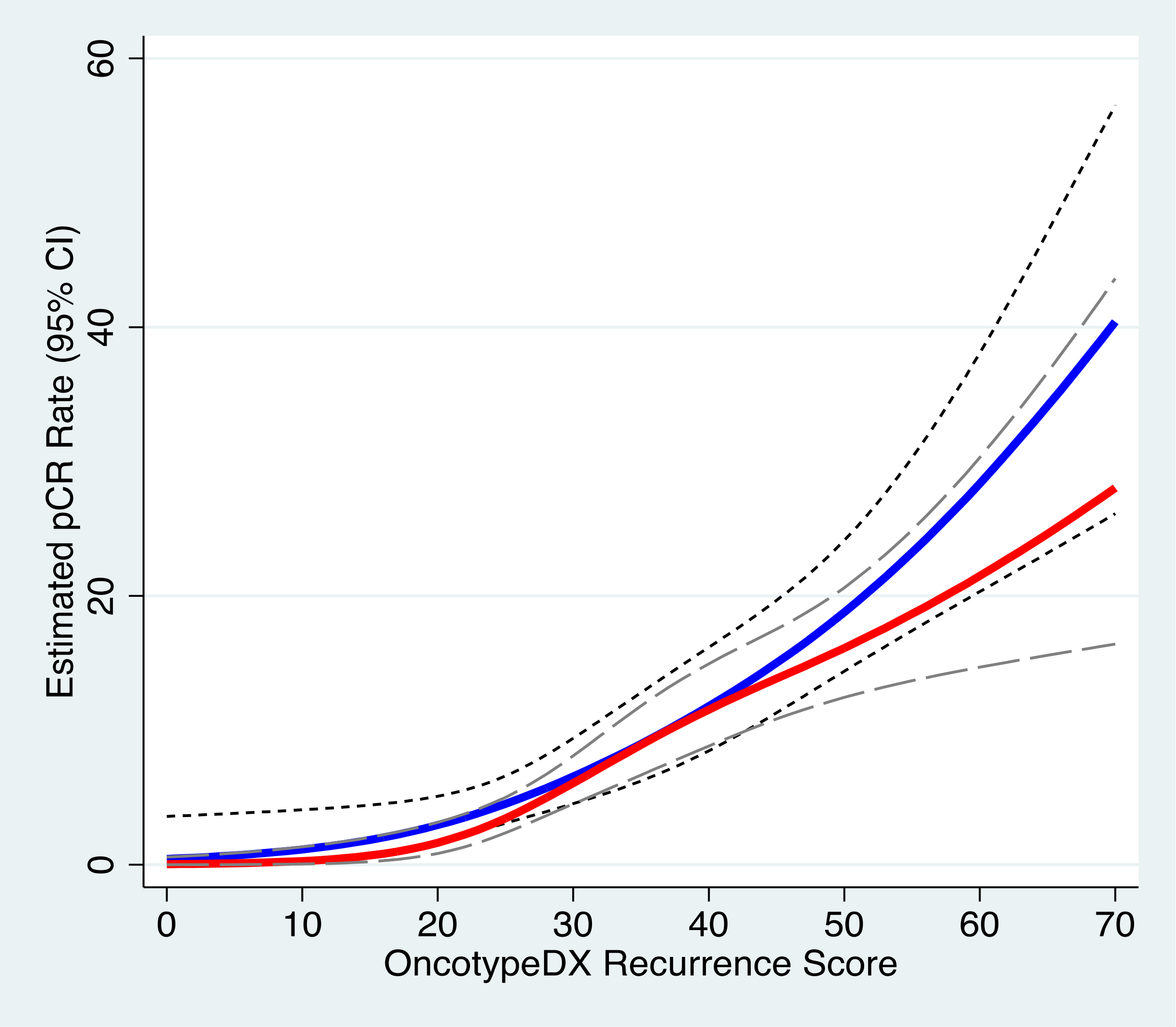

The blue line indicates the estimated pCR rates in patients who were 50 years or younger, while the red line indicates the estimated pCR rates in patients who were above 50 years. Dotted lines indicate 95% confidence interval.
Abbreviations: pCR, pathologic complete response.

Supplementary Table 3. Characteristics of early-stage breast cancer patients who received neoadjuvant chemotherapy by MammaPrint result

	**MammaPrint result**		
**Variable**	**Low risk** (n=208), No. (row %)	**High risk** (n=1,141), No. (row %)	***P* value** [a]
**Age at diagnosis**, mean (SD)	53.5 (11.8)	53.2 (12.0)	.70
**Race/Ethnicity**			
White	153 (15.3)	849 (84.7)	.58
Black	19 (12.8)	129 (87.2)	
Asian	13 (20.6)	50 (79.4)	
Hispanic	20 (18.0)	91 (82.0)	
**Year of diagnosis**			
2010-2011	18 (22.2)	63 (77.8)	.12
2012-2013	34 (15.5)	185 (84.5)	
2014-2015	42 (14.5)	247 (85.5)	
2016-2017	48 (18.8)	208 (81.3)	
2018-2019	66 (13.1)	438 (86.9)	
**Charlson-Deyo Score**			
0	186 (15.6)	1,007 (84.4)	.63
≥1	22 (14.1)	134 (85.9)	
**Histologic type**			
Ductal	146 (12.9)	982 (87.1)	<.0001
Lobular	36 (34.0)	70 (66.0)	
Other	26 (22.6)	89 (77.4)	
**PR status**			
Negative	24 (5.1)	445 (94.9)	<.0001
Positive	183 (20.9)	693 (79.1)	
**ER status**			
Negative	11 (3.6)	296 (96.4)	<.0001
Positive	197 (18.9)	845 (81.1)	
**HER2 status**			
Negative	181 (16.4)	922 (83.6)	.03
Positive	27 (11.0)	219 (89.0)	
**AJCC stage group**			
I	43 (15.2)	240 (84.8)	.59
II	122 (14.9)	699 (85.1)	
III	43 (17.6)	202 (82.5)	
**AJCC clinical T stage**			
cT1	40 (14.7)	233 (85.4)	.14
cT2	112 (14.3)	669 (85.7)	
cT3	46 (20.6)	177 (79.4)	
cT4	10 (14.1)	61 (85.9)	
**AJCC clinical nodal status**			
Negative (cN0)	116 (14.1)	707 (85.9)	.08
Positive (cN1+)	92 (17.6)	431 (82.4)	
**Tumor grade**			
1	37 (36.6)	64 (64.4)	<.0001
2	127 (21.6)	461 (78.4)	
3	33 (5.4)	576 (94.6)	
**Days from initial diagnosis to start of chemotherapy**, median (IQR)	37 (27-53)	40 (28-54)	.37
**Receipt of neoadjuvant hormone therapy**			
No	138 (17.5)	652 (82.5)	.06

Yes	39 (23.8)	125 (76.2)	

Abbreviations: RS, recurrence score; SD, standard deviation; PR, progesterone receptor; ER, estrogen receptor; HER2, human epidermal growth factor receptor 2; AJCC, American Joint Committee on Cancer; IQR, interquartile range.

[a] *P* values were calculated using Student's t or Wilcoxon rank-sum tests for continuous data and Pearson's Chi-Squared tests for categorical data.

Supplementary Table 4. Association between type of surgery and multigene assay results among early-stage breast cancer patients who received neoadjuvant chemotherapy

	**Type of surgery**			**Logistic regression**	
**Variable**	**Lumpectomy** No. (row %)	**Mastectomy** No. (row %)	***P* value**	**AOR (95% CI)**	***P* value**
**Oncotype DX recurrence score** [a]					
Continuous, Mean (SD)	31.2 (14.3)	26.6 (14.2)	<.0001	1.21 (1.12-1.31) [b,c]	<.0001
Categorical [d]					
0-25	313 (46.5)	360 (53.5)	<.0001	1.0 (reference)	
26-100	606 (64.3)	337 (35.7)		1.83 (1.48-2.25) [c]	<.0001
**MammaPrint result**					
Any early-stage breast cancer					
Low risk	82 (51.9)	76 (48.1)	.006	1.0 (reference)	
High risk	565 (63.4)	326 (36.6)		1.46 (1.02-2.08) [c]	.037

Abbreviations: SD, standard deviation; AOR, adjusted odds ratio; CI, confidence interval; PR, progesterone receptor; ER, estrogen receptor; HER2, human epidermal growth factor receptor 2.

[a] The Oncotype DX cohort included patients with hormone receptor (HR)-positive/HER2-negative stage I-III disease.

[b] AOR per 10 unit increase in Oncotype DX score.

[c] adjusted for clinical T and N stages.

[d] Oncotype DX recurrence score was dichotomized per the TAILORx trial cutoff.
